# Predictors of Complications in Prophylactic Mastectomy and Direct-to-Implant Breast Reconstruction: A Retrospective, Single-Center Study

**DOI:** 10.3390/jcm15052071

**Published:** 2026-03-09

**Authors:** Anna Wiesmeier, Lukas Prantl, Florian Zemann, Silvan Eisenmann, Vanessa Brebant, Dmytro Oliinyk, Philipp Unbehaun, Sophia Diesch, Marc Ruewe, Alexandra M. Anker

**Affiliations:** 1 Department of Plastic, Reconstructive and Hand Surgery, University Hospital Regensburg, Franz-Josef-Strauss-Allee 11, 93053 Regensburg, Germanyvanessa.brebant@ukr.de (V.B.);; 2 Center of Clinical Studies, University Hospital Regensburg, Franz-Josef-Strauss-Allee 11, 93053 Regensburg, Germany

**Keywords:** gene mutation, breast cancer, prophylactic mastectomy, subcutaneous mastectomy, complication rates

## Abstract

**Background/Objectives:** Prophylactic mastectomy can significantly reduce the risk of breast cancer in patients carrying gene mutations such as BRCA1 and BRCA2. Patients who opt for breast removal are offered tailored reconstructive options based on their medical history and prior treatments, and in these often young patients with limited autologous tissue reserves, implant-based reconstruction is frequently the option of choice. Complication rates of these procedures are relatively high and account for up to 30%. Subcutaneous mastectomy with primary implant reconstruction carries risks such as hematoma, seroma, skin necrosis, necrosis of the nipple–areola complex, and wound healing issues, which may necessitate revision surgery. This university-center retrospective analysis aims to improve outcomes by identifying patient- and surgery-related risk factors associated with postoperative complications in allogenic breast reconstruction following subcutaneous mastectomy. **Methods:** We analyzed 61 female patients and 122 breasts who underwent primary implant-based reconstruction after skin- or nipple-sparing subcutaneous mastectomy over three years between January 2021 and December 2023. Demographic and surgical variables were systematically collected and analyzed. **Results:** The mean patient age was 41.5 ± 10.3 years. A total of 13% of patients were active smokers, and 1.6% had diabetes mellitus. Overall, skin flap necrosis occurred in 27.9% of patients (22.1% of breasts), wound healing disorders in 19.7% of patients, wound infections in 9.8%, and revision surgery in 18.0%. A history of pregnancy was associated with skin flap necrosis (OR 10.07, 95% CI 1.79–190.06; *p* = 0.032); however, this finding must be interpreted with caution due to limited statistical power and model instability. **Conclusions:** This investigation revealed clinically relevant patterns suggesting potential risk factors for wound healing disorders and skin necrosis. Prospective studies are planned to further substantiate these findings and to help reduce overall complication rates associated with the procedure.

## 1. Introduction

Breast cancer is the most common cancer in women worldwide. It is estimated that at least 5–10% of all breast cancers occur due to genetic predisposition [1]. With an estimated prevalence of 1 in 300–500 BRCA1 and -2 mutations in the general population, around 3–4% of all breast cancers are caused by mutations in these specific genes [2]. Other gene mutations, such as those in the ATM, PALB2 and CHEK2 genes, are also associated with an increased risk of tumor formation, including breast cancer [3]. Following genetic diagnostics, affected patients receive detailed counseling. In addition to intensified early detection programs, risk-reducing surgical options are offered to genetically predisposed patients, who are often young and in good general health. Prophylactic subcutaneous mastectomy can reduce the risk of breast cancer by 90% [3,4,5].

During this surgery, complete resection of the mammary gland is performed, either leaving the complete skin envelope and nipple–areola complex intact (nipple-sparing mastectomy), or resecting the nipple (with or without the areola) but preserving the residual skin envelope (skin-sparing mastectomy). This procedure is usually performed bilaterally in healthy mutation carriers, or unilaterally in patients undergoing surgery for cancer in the opposite breast [6].

Taking into account the average age at which cancer occurs in the presence of a gene mutation, the optimal time to perform a bilateral prophylactic mastectomy in healthy mutation carriers is at the age of 25–30 years [7]. This young age underscores the importance of achieving an optimal aesthetic outcome through prophylactic surgery. In young, slender patients with no suitable donor sites for free flap reconstruction, implant reconstruction is often the preferred option. Moreover, this procedure can be carried out concurrently with the mastectomy. It is faster than free flap surgery and spares wounds or defects in the donor area. Around 80% of all breast reconstructions are implant-based [8].

Complication rates for implant-based reconstruction following mastectomy procedures, however, account for up to 30% [9,10]. Typical complications include hematoma, increased seroma formation, infections, skin necrosis, necrosis of the nipple–areola complex (NAC), and wound healing disorders. In the worst-case scenario, a revision procedure is necessary, which eventually may even result in implant loss and reconstructive failure. Long-term complications such as capsular contracture and breast implant-associated anaplastic large cell lymphoma (BIA-ALCL) must also be considered [8,11,12,13].

To fully realize the concept of a “risk-reducing mastectomy,” and minimize postoperative complications, the aim of this study was to characterize the typical patient cohort undergoing subcutaneous mastectomy and direct allogenic reconstruction on the one hand, and to investigate potential risk factors for complications in primary implant-based breast reconstruction following subcutaneous mastectomy on the other.

## 2. Materials and Methods

This study was conducted with the approval of the Ethics Committee of the University of Regensburg (reference 24-3727-104). A retrospective review of medical records was performed between January 2021 and December 2023 at the Department of Plastic, Hand and Reconstructive Surgery, Caritas Hospital St. Josef, Regensburg, Germany.

**Figure 1:**
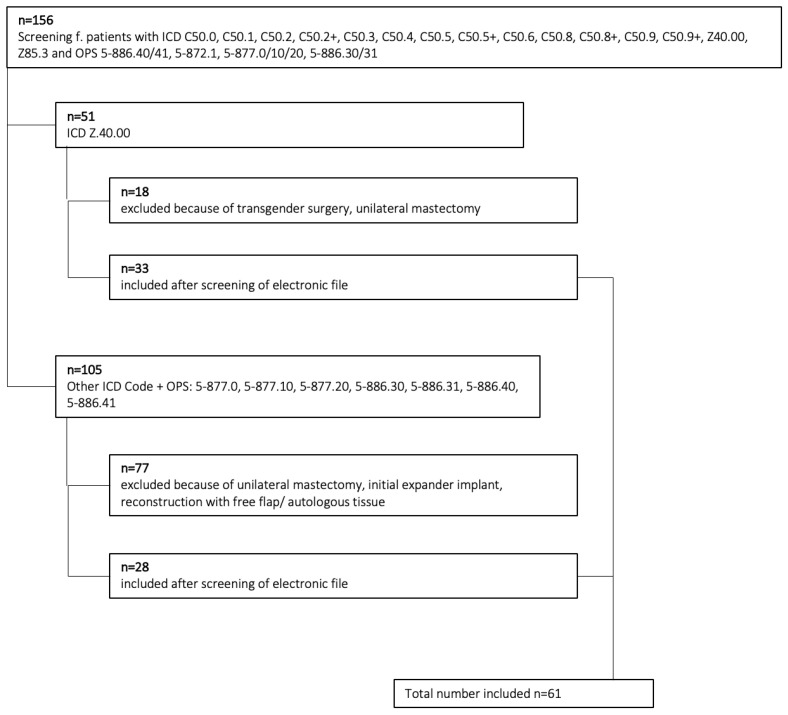
Prisma diagram of selection process.

An initial screening was performed to identify suitable ICD and OPS codes. ICD 50 stands for a malignant neoplasm of the breast, followed by the number for the respective location of the tumor within the breast (ICD: C50.0, C50.1, C50.2, C50.2+, C50.3, C50.4, C50.5, C50.5+, C50.6, C50.8, C50.8+, C50.9, C50.9). Z40.00 stands for “Prophylactic surgery of the breast due to increased risk of malignant neoplasm” and Z85. 3 for “Personal history of malignant neoplasm of the breast”. The OPS Code 5-886.XX stands for mastectomy, the last two digits code the extent of resections of the skin envelope (0.30 “without removal of the nipple–areola complex”, 0.31 “with removal of the nipple–areola complex”, 0.40 “skin-sparing mastectomy”, 0.41 “skin- and nipple-sparing mastectomy”). OPS 5-872.1 codes “Breast reconstruction using an implant”; 5-877.0/10/20 are the OPS codes for “Plastic surgery of the breast, not otherwise specified”, “Plastic surgery with local tissue rearrangement”, and “Plastic surgery with complex reconstruction”. Of the 156 identified hits, 51 had the ICD code Z40.00 (prophylactic surgery for risk factors related to malignant neoplasms: prophylactic breast surgery), and 33 suitable patients were included after dedicated file review. Further ICD codes with matching OPS codes for primary breast reconstruction resulted in 105 cases, of which 28 patients were finally included after careful manual selection (Table 1). In total, 61 patients undergoing subcutaneous nipple- or skin-sparing mastectomy with immediate reconstruction using breast implants were included (Figure 1).

Patient records were reviewed for demographic information, relevant medical and surgical history, details of the mastectomy surgery and breast implants, and any subsequent complications. Potential risk factors, including nicotine use and prior radiotherapy, were also documented. The selection of evaluated risk factors was informed by a comprehensive review of current literature and refined according to the expert consensus of our team, comprising over 20 plastic surgeons with extensive clinical experience in breast reconstruction. All data were compiled in tables and subjected to statistical analysis.

### Statistical Analyses

Data are presented descriptively by using mean (SD) for continuous and absolute (relative) frequencies for categorical data. For patient-level analyses, each individual was included only once. Associations between potential clinical risk factors and the occurrence of skin necrosis were assessed using univariate logistic regression models with a binomial distribution and logit link function, and results are reported as odds ratios (ORs) with 95% confidence intervals (CIs). To evaluate potential surgical risk factors while accounting for repeated measurements within patients, multilevel logistic regression models were fitted with a random intercept for patient. These generalized linear mixed-effects models allowed for clustering of observations at the patient level and were likewise specified with a binomial distribution and logit link. Due to the limited number of events, all analyses were conducted univariate. *p* < 0.05 was determined to be significant. All statistical analyses were performed using R, version 4.5.1 (R Foundation for Statistical Computing, Vienna, Austria), employing the lme4 package for mixed-effects models and base R functions for logistic regression.

## 3. Results

A total of 61 patients were included in the study after applying the inclusion and exclusion criteria. This corresponds to n = 61 at the patient level and n = 122 when considering each breast.

### 3.1. Demographic and Baseline Data

The average age at the time of surgery was 41.5 (SD 10.3) years. In the purely prophylactic group, it was 39.1 (SD 10.1) years. The average BMI of the entire group (including both prophylactic and therapeutic/curative mastectomies) was 24.2 (SD 4.2) kg/m^2^, and in the solely prophylactic mastectomy group it was 22.9 (SD 3.2) kg/m^2^.

Of all patients included, eight patients were smokers, one patient had a history of diabetes mellitus and four (6.6%) had previously undergone radiation therapy to the chest area for other reasons (n = 3 breast cancer in earlier years, n = 1 Hodgkin’s disease). At the time of surgery, 36 patients (59.0%) had already given birth to one or more children (Table 2).

A BRCA1 mutation was detected in 25 patients (41%) and a BRCA2 mutation in 16 patients (26.2%). Other mutations detected were CHEK2 (n = 3), PALB2 (n = 5), ATM (n = 1), and BARD1 (n = 1). Ten patients had no detectable mutation and underwent mastectomy for other reasons (fear of cancer, family history of cancer, etc.). Of the patients included in the study, 28 (45.9%) had no active breast cancer at the time of surgery and therefore underwent bilateral prophylactic mastectomy. Thirty-one patients (50.8%) were diagnosed with breast cancer, and two (3.3%) suffered from DCIS. Among these 33 breast cancer patients, nine had previously undergone breast-conserving surgery; three of these also underwent axillary lymph node dissection, and five underwent sentinel node biopsy. Twenty-four (39.3%) patients received neoadjuvant or adjuvant chemotherapy, ten (16.4%) received antihormone therapy, and two (3.3%) received antibody therapy. Postoperative radiotherapy was administered to eight patients (13.1%) (Table 3).

### 3.2. Surgical Data

**Figure 2:**
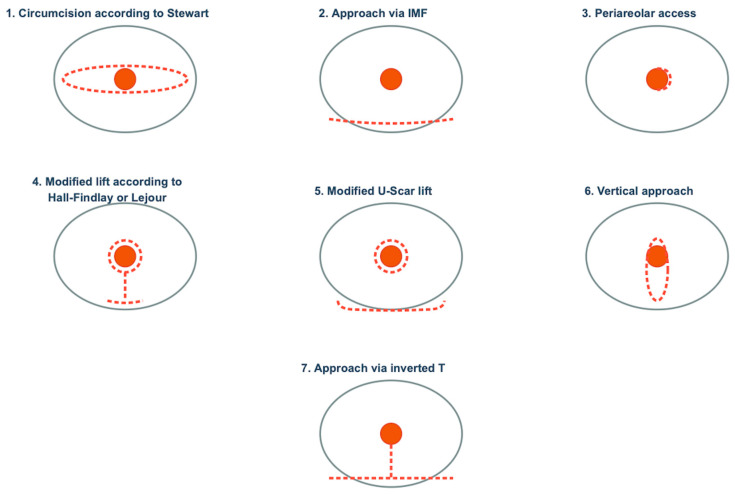
Operative approaches for subcutaneous mastectomy.

Following mastectomy, primary breast reconstruction was performed using implants according to the inclusion criteria in all patients. Four out of 122 implants (3.3%) were placed in prepectoral position, while 118 (96.7%) were placed subpectorally. In 34 breasts (27.9%) the NAC was resected, while in 12 (9.8%) only the nipple was resected while preserving the areola. In all other breasts the nipple and areola were completely preserved (n = 76). In 52 breasts (42.6%), the surgical approach was via the IMF, in 31 (25.4%) mastectomy was performed via a Stewart spindle, in 33 (27.0%) via an inverted T. Two breasts each underwent mastectomy via U-Scar, periareolar or vertical approach (1.6% each). Figure 2 shows the different types of surgical incisions. An ADM was used to close the implant pocket in 73 breasts (59.8%), a musculofascial flap in 16 breasts (13.1%), a dermal flap in 23 breasts (18.9%), and a combination of muscle flap and dermal flap in 10 breasts (8.2%). In all four cases of prepectoral implant placement, the caudal border was formed by a fasciomuscular flap. In those four breasts, polyurethane textured implants were used (Microthane, POLYTECH Health & Aesthetics GmbH, Dieburg, Germany) (Table 4).

### 3.3. Risk Factors

**Figure 3:**
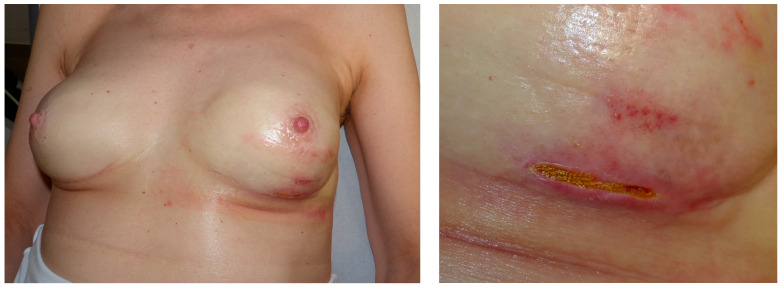
Impairment of wound healing treated conservatively.

**Figure 4:**
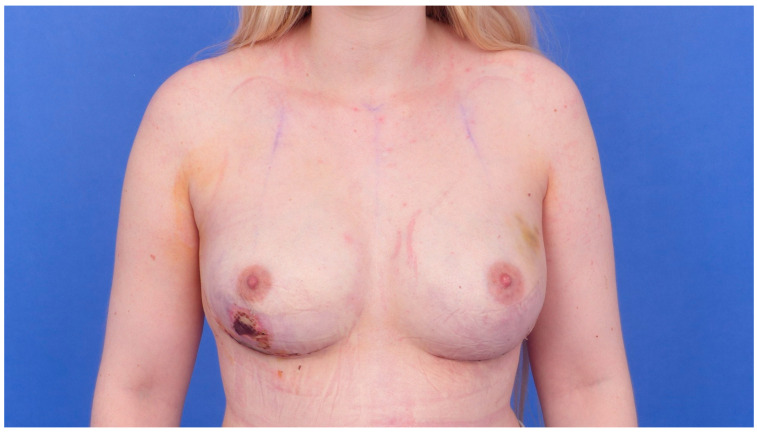
Necrosis of the right breast with indication for necrosectomy, removal of implant and placement of a temporary tissue expander.

The following potential patient- and surgery-related risk factors for complications were analyzed (Table 5):

The evaluation of complications after mastectomy shows necrosis of the skin envelope in 17 patients and 27 breasts. In twelve patients and 23 breasts, wound healing disorders occurred (n = 11 on both sides, n = 1 on one side only). Eleven patients and 16 breasts underwent revision surgery due to complications (Table 6). Examples are shown in Figure 3 and Figure 4.

A comparison was made between the complication rates in patients with diagnosed breast cancer and those who underwent purely prophylactic mastectomy. The overall complication rate accounted for 66.67% (n = 22 of n = 33) at the patient level and 60% (n = 21 of n = 35) at the breast level for patients diagnosed with breast cancer. For prophylactic mastectomies, the overall complication rate was slightly lower with 64.29% (n = 18 of n = 28) at the patient level and 51.71% (n = 45 of n = 87) at the breast level. There is no significant difference between the two groups (patient level: *p* = 1.0, OR 1.11; breast level: *p* = 0.429, OR 1.40 using Fisher’s exact test).

At the level of patient-related risk factors predisposing to postoperative necrosis, a previous pregnancy showed a significant result (*p* = 0.032) with an OR of 10.07 (95%-CI, 1.79–190.06).

No significant risk factors regarding BMI or previous tumor therapies for the development of skin necrosis were identified at the patient level (Table 7).

Significance testing did not reveal any clear risk factors in the analysis of the surgery-related data. Variables such as the incision type, NAC preservation, resection weight, and implant volume showed no apparent association with the development of necrosis. The results are summarized in Table 8.

After removal of the mammary gland, the specimen was processed histopathologically. The results are listed in Table 9.

## 4. Discussion

The aim of this study was to characterize the patient population typically undergoing subcutaneous nipple- or skin-sparing mastectomy with direct-to-implant reconstruction and to identify potential risk factors for complications in this sensitive patient group.

Subcutaneous nipple- or skin-sparing mastectomies can be performed both therapeutically in patients with diagnosed breast cancer and prophylactically in high-risk individuals carrying genetic mutations such as BRCA1 or BRCA2.

The latter cohort typically includes young women with a slender body and insufficient donor tissue for autologous breast reconstruction, making implant-based reconstruction the preferred method.

Current literature and guidelines on prophylactic mastectomies consider an age of 25–30 years to be the optimal time frame for surgery [6,7]. At 39 years, the average age of our cohort was above this recommended age for performing mastectomy, but comparable to the cohorts from other studies [13,14]. Additionally, heterogeneity in national screening protocols and the availability and timing of genetic testing for hereditary breast cancer risk (e.g., BRCA1/2) may influence the age at which individuals are identified as high-risk and elect for prophylactic surgery. Variations in these preventive pathways across different healthcare settings could partly explain why our cohort’s average age exceeds the typically recommended window [15].

In the prophylactic mastectomy group, we had an average BMI of 22.94 kg/m^2^, indicating that the women were of normal weight. Only one of the 61 patients had a known history of diabetes mellitus, and eight were smokers. No other relevant pre-existing conditions were found in the medical histories. The observed patient characteristics are consistent with those reported in other cohorts in the literature [16]. In addition to oncological prophylaxis, these patients have particularly high expectations regarding aesthetic outcomes, as they often have a well-formed and aesthetically pleasing breast prior to the “risk-reducing” procedure.

Although implant-based reconstruction is associated with shorter operative times and faster postoperative recovery compared to autologous reconstruction, the procedures remain technically demanding. The current literature describes complication rates between 10 and 30% [9,10]. These numbers correspond to our results of 20% necrosis and 10% revision surgery rates of all reconstructed breasts.

A key focus of our analysis was the identification of risk factors for wound healing problems, particularly skin flap perfusion issues, which represent one of the most common complications in subcutaneous mastectomy with direct-to-implant reconstruction.

Initially, potential patient-related risk factors were empirically defined by expert consensus, and the literature was reviewed for relevant risk factors.

Obesity is associated with increased risks of infections, wound complications, and circulatory disorders following implant-based reconstruction [17]. This also applies for surgical procedures in general [18,19].

In addition, nicotine abuse is known to be a risk factor for postoperative complications. This was also demonstrated in a large cohort study that examined the effects of nicotine after plastic surgery procedures. It has been shown that active smoking is strongly associated with complications. Significantly higher rates of tissue necrosis were observed, and revision surgery was required more often [19,20].

Preoperative therapies for already diagnosed breast cancer were also recorded as risk factors. Previous or postoperative radiation is known to damage soft tissue and cause poor skin quality, which promotes wound healing disorders, circulatory disorders, infections, and capsular fibrosis [21]. Adjuvant endocrine therapy (e.g., tamoxifen or aromatase inhibitors) has been associated with increased postoperative infections in some studies of implant-based reconstruction, but effects on skin flap necrosis and overall wound healing remain uncertain [22,23]. Although neoadjuvant or adjuvant chemotherapy can promote infections due to immune deficiency, it is not considered an independent risk factor for wound healing disorders or skin necrosis [17,24]. Antibody therapies also show no clear tendency toward increased complications [25].

While the abovementioned potential risk factors could not be confirmed in this study for the specific patient cohort undergoing subcutaneous mastectomy and direct-to-implant reconstruction, there was a statistically significant correlation between a previous pregnancy and the development of skin flap necrosis (OR 10.07; 95% CI 1.79–190.06; *p* = 0.032). To our knowledge, this association has not been previously described. A plausible explanation could be long-term alterations in microvascular perfusion, tissue elasticity, or skin architecture following pregnancy-related breast changes and weight fluctuations, potentially affecting skin flap viability. However, no clear correlation can be established in a small number of cases, which is why further clinical studies involving larger numbers of cases are necessary.

Interestingly, complication rates did not differ significantly between therapeutic and prophylactic mastectomies. This is particularly noteworthy given that in 8 of 33 cases postoperatively and in 2 of 33 cases preoperatively, patients with cancer had undergone radiotherapy, which impairs tissue quality and is known to be a risk factor for postoperative complication as mentioned earlier. Nevertheless, our results align with systematic meta-analytic evidence showing no significant difference in necrosis, seroma, or capsular contracture rates between therapeutic and prophylactic reconstructions, even if other complications such as infection and explantation rates may differ [10].

No significant associations were observed for other established patient-related risk factors such as BMI, genetic predisposition, prior oncological therapies, or smoking. While traditional risk factors including elevated BMI, smoking, diabetes mellitus, and radiotherapy have been repeatedly identified as independent predictors of flap necrosis due to detrimental effects on microvascular circulation and wound healing, the absence of significance in our cohort may reflect the relatively low prevalence of these factors within our generally healthy, well-defined and highly specific patient cohort.

In addition to patient-related risk factors, surgery-related risk factors were examined.

Studies comparing prepectoral and subpectoral implant placement show statistically higher seroma rates and increased rippling with prepectoral implants. Submuscular implants, on the other hand, cause animation of the pectoralis muscle [17]. However, there is no significant difference in terms of wound healing disorders, infections, hematomas, skin necrosis, revision rates, capsular fibrosis, or implant rotation [26]. A retrospective analysis from 2025 examined breast reconstruction using prepectoral polyurethane (PU) implants, considering possible risk factors and complications. Of the 317 breast reconstructions, 6.3% required revision surgery due to major complications, such as bleeding, infection, skin necrosis, and wound dehiscence. In some cases, the edges of the implants were visible in the décolletage area, or the implants rippled, indicating the need for additional lipofilling. However, prepectoral implant placement requires a well-perfused and stable skin envelope; thus, each case must be evaluated individually. This study does not specifically compare necrosis rates or circulatory disorders between prepectoral and subpectoral implant reconstruction. Further studies are needed in this area [17]. Our study did not demonstrate significant differences based on implant plane, the small number of prepectoral cases, however, limits interpretation.

Increased body weight and high mammary gland resection weight in macromastia have been shown to be risk factors for skin and NAC necrosis. High-volume stress on the skin envelope due to large implants or overly filled expanders has also been identified as a risk factor for impaired blood circulation of the skin envelope [27,28,29]. Based on these findings, this study analyzed body weight, BMI, and the ratio of implant weight to resection weight as risk factors. “Overfilling” the skin envelope can result in perfusion disorders due to increased tension and pressure. Our study did not reveal any significant results with regard to the complications that occurred. However, based on the literature to date, it is possible that subclinical perfusion disorders occur. Further analysis and examination during surgery could involve measuring layer thickness using ultrasound and assessing skin envelope perfusion using indocyanine green fluorescence (ICG). Intraoperative ICG angiography has been shown to assess the perfusion of mastectomy skin flaps in real time. This allows poorly perfused areas to be identified and potentially resected during surgery, which has been shown to correlate with reduced rates of flap necrosis and reoperation [30,31,32]. The use of ultrasound to measure mastectomy skin flap thickness during surgery has been described as a complementary method to clinical assessment, and several narrative reviews consider such instrumental evaluations alongside perfusion imaging to help predict ischemic complications [31,33]. Preoperative imaging using an MRI scan can also help identify the correct dissection plane between glandular and subcutaneous fatty tissue. This layer’s thickness can be measured prior to mastectomy and used as a guide during surgery [33]. A multimodal approach and established standards for the use of technical measurement methods are necessary in order to prevent skin necrosis as far as possible.

Furthermore, the choice of incision is a key element in surgical planning as it directly affects the perfusion of mastectomy skin flaps and the risk of ischemic complications. Previous studies have demonstrated that periareolar and circumareolar incisions disrupt critical vascular pathways, resulting in higher rates of NAC ischemia. In contrast, IMF incisions generally demonstrate more favorable perfusion outcomes and lower complication rates [34,35]. Other studies show a higher rate of NAC necrosis with periareolar incisions and higher rates of skin necrosis with IMF access compared to radial incisions [36,37].

In prophylactic mastectomies, it is often feasible to preserve the skin envelope and the NAC, allowing direct-to-implant reconstruction through either an IMF or periareolar incision. While no single incision technique is universally superior, the current evidence base supports an individualized approach that considers patient anatomy, degree of ptosis, breast size and reconstructive goals. In therapeutic settings, however, oncological safety dictates the choice of incision: for example, women with breast cancer who are mutation carriers frequently require NAC resection using patterns such as Stewart spindle incisions. Additional techniques, such as mastopexy approaches, enable the safe resection of excess skin and its reduction in patients with macromastia or ptosis [38]. Our study shows no significant differences in complication rates with regard to the chosen surgical approach or whether the NAC is preserved or resected. The absence of significant associations in our analysis is likely due to limited statistical power given the modest sample size and relatively low overall number of necrosis events (n = 27 of 122 breasts).

After the mammary gland has been removed, a histopathological analysis of the tissue was performed. If breast cancer is already known to be present, the final tumor stage and completeness of resection are of particular interest. The tumor’s response to any previous chemotherapy can also be determined. Studies have shown that, in cases of purely prophylactic mastectomy, carcinoma or preliminary stages can be detected histopathologically in over 11% of cases, even when previous imaging diagnostics did not reveal any abnormalities [39,40]. The histopathological result is essential for determining further oncological therapy and plastic surgery. Our histopathological findings, consistent with other reports, emphasize that occult carcinoma or precursor lesions can be detected in prophylactic specimens in a notable minority of cases, underscoring the importance of routine pathology even when imaging appears normal.

## 5. Limitations

While this study allowed for a detailed characterization of patients undergoing subcutaneous mastectomy with primary implant-based reconstruction and the analysis of patient- and surgery-related risk factors for skin flap necrosis and overall complications, several limitations must be acknowledged. Due to the narrow and well-defined cohort, the study is limited by the relatively small number of included breasts, which inevitably restricts statistical power. However, this is offset by the high degree of cohort homogeneity, which reduces clinical heterogeneity and strengthens internal validity by minimizing variability related to indication, surgical technique and patient characteristics. Additionally, the low number of observed complication events limits the feasibility of robust multivariate analyses. Notably, these low event rates are consistent with complication frequencies reported in the existing literature, suggesting that the findings reflect contemporary clinical practice. The overall complication rate should be interpreted with caution as the patient population investigated represents a particularly sensitive group with high aesthetic expectations, for whom even minor complications may have disproportionate clinical relevance. Therefore, the overarching goal should be to further reduce complication rates to an absolute minimum, beyond confirming favorable outcomes. The retrospective design and reliance on univariate analyses further limit causal inference and adjustment for residual confounding factors. Nevertheless, the study provides valuable insight into this clearly defined patient group and highlights the need for future multicenter, prospective investigations with standardized complication definitions and comprehensive risk stratification to improve outcome optimization in this challenging population.

## 6. Conclusions

Our results confirm that subcutaneous nipple- or skin-sparing mastectomy with direct-to-implant reconstruction is performed predominantly in young, healthy women. Nevertheless, overall complication rates remain persistently high, reaching up to 30%. Prior pregnancy emerged as a novel potential risk factor for skin flap necrosis, meriting further investigation. Traditional risk factors remain clinically relevant, and thorough preoperative risk assessment should continue to guide patient counseling and surgical planning. Larger prospective studies are needed to better delineate risk factors and optimize outcomes in both therapeutic and prophylactic mastectomy populations.

## Figures and Tables

**Table 1:** In- and exclusion criteria.

Inclusion Criteria	Exclusion Criteria
Patients with indication for bilateral subcutaneous mastectomy	Two-staged procedure, e.g., with temporary insertion of an expander
Diagnosed breast cancer	Reconstruction with autologous tissue
Evidence of a genetic predisposition	Patients <18 years of age
Primary implant-based breast reconstruction	Patients in need of care/particularly vulnerable patients

**Table 2:** Medical history.

	Number (n = x Out of n = 61)
Smoking	8 (13.1%)
Diabetes mellitus	1(1.6%)
Radiation for other reason	4 (6.6%)
History of pregnancy	36 (59.0%)

**Table 3:** Indications for mastectomy and further therapies.

	Number (n = x Out of n = 61)
Indication for mastectomy	
Prophylactic mastectomy (bilateral, no active breast cancer)	28 (45.9%)
Mastectomy for existing breast cancer	31 (50.8%)
Mastectomy for DCIS	2 (3.3%)
Previous surgeries	
Breast-conserving surgery before mastectomy	9 (14.8%)
Axillary lymph node dissection	3 (4.9%)
Sentinel lymph node biopsy	5 (8.2%)
Adjuvant or neoadjuvant therapies	
Chemotherapy (neo-/adjuvant)	24 (39.3%)
Antihormonal therapy	10 (16.4%)
Antibody therapy	2 (3.3%)
Postoperative radiation	8 (13.1%)
Genetic mutations	
BRCA1 mutation	25 (41.0%)
BRCA2 mutation	16 (26.2%)
CHEK2 mutation	3 (4.9%)
PALB2 mutation	5 (8.2%)
ATM mutation	1 (1.6%)
BARD1 mutation	1 (1.6%)
No mutation (other reasons for mastectomy)	10 (16.4%)

Abbreviations: DCIS (Ductal Carcinoma In Situ), BRCA1/2 (Breast Cancer Susceptibility Genes), CHEK2 (Checkpoint Kinase 2), PALB2 (Partner and Localizer of BRCA2), ATM (Ataxia Telangiectasia Mutated), BARD1 (BRCA1-Associated RING Domain 1).

**Table 4:** Surgical data.

	Number (n = x Out of n = 122 Breasts)
**Plane of placement**	
Prepectoral implants	4 (3.3%)
Subpectoral implants	118 (96.7%)
**Treatment of NAC**	
NAC resection	34 (27.9%)
Nipple resection, areola preservation	12 (9.8%)
**Incision**	
IMF approach	52 (42.6%)
Stewart approach	31 (25.4%)
Inverted T approach	33 (27.0%)
Periareolar approach	2 (1.6%)
U-Scar approach	2 (1.6%)
Vertical approach	2 (1.6%)
**Pocket closure/caudal stability**	
ADM	73 (59.8%)
Muscle/fascia flap	16 (13.1%)
Dermis flap	23 (18.9%)
Combination of dermis and muscle flap	10 (8.2%)

Abbreviations: NAC (nipple–areola complex), IMF (inframammary fold), ADM (acellular dermal matrix).

**Table 5:** Patient-related and surgery-related risk factors.

Patient-Related Risk Factors
BMI (Body Mass Index)
Adjuvant therapies: ° Radiation ° Chemotherapy ° Antihormonal treatment ° Antibody therapy
Tumor characteristics
Genetic predisposition
Pregnancy history
Surgery-related risk factors
Incision type (inframammary fold [IMF] vs. Stewart vs. inverted T). Vertical, periareolar, and U-scar incisions were excluded due to insufficient sample size
NAC management (areola and nipple resection/preservation, nipple-only resection/preservation, NAC skin graft)
Resection weight of mammary gland tissue
Implant volume to resection weight ratio (categorized as implant volume—resection weight = <−50 mL, −50 to 50 mL, >50 mL)

**Table 6:** Complication rates.

Complication	Number (n = x Out 61 Patients)	Number (n = x Out of 122 Breasts)
Necrosis	17 (27.9%)	27 (22.1%)
Impairment of wound healing	12 (19.7%)	23 (18.9%)
Revision surgery	11 (18.0%)	16 (13.1%)

**Table 7:** Results of univariate analysis of patient-related risk factors (n = 61 patients, OR: odds ratio, 95%-CI: 95% confidence interval, * = significant result).

Variable	OR (95%-CI)	*p* Value
BMI	1.01 (0.88–1.15)	0.861
Radiation	3.08 (0.65–14.78)	0.147
Chemotherapy	1.56 (0.49–4.88)	0.445
Antihormone therapy	0.60 (0.08–2.75)	0.547
Antibody therapy	2.69 (0.10–70.73)	0.494
Genetic predisposition	4.11 (0.68–79.16)	0.197
History of pregnancy	10.07 (1.79–190.06)	0.032 *

**Table 8:** Results of univariate analysis of surgery-related risk factors (n = 122 breasts, OR: odds ratio, 95%-CI: 95% confidence interval).

Variable	OR (95%-CI)	*p* Value
Incision		
IMF	reference	reference
Stewart	0.10 (0.00–179)	0.542
Inverted T	1.46 (0.03–64)	0.843
NAC preservation	1.46 (0.03–64)	0.265
Implant volume—resection weight ratio		
+/−50	reference	reference
<−50	1.15 (0.02–61)	0.944
>+50	1.33 (0.04–45)	0.873
Weight of resected tissue	1.00 (1.00–1.01)	0.658

Abbreviations: IMF (inframammary fold), NAC (nipple–areola complex).

**Table 9:** Histopathological findings.

Benign Findings	Number n= x Out of n = 122 Breasts
Fibrosis	25 (20.5%)
Mastopathia	10 (8.2%)
Apocrine metaplasia	8 (6.6%)
Adenosis	5 (4.1%)
UDH	4 (3.28%)
Fibroadenoma	3 (2.5%)
Papilloma	2 (1.6%)
Microcalcifications	3 (2.5%)
ADH	1 (0.8%)
**Malignant findings**	Number n = x out of n = 122
Complete remission after chemotherapy	20 (16.4%)
Residual DCIS	2 (1.6%)
Newly diagnosed carcinoma	1 (0.8%)
LIN	2 (1.6%)
Complete resection of known carcinoma	8 (6.6%)

Abbreviations: UDH (Usual Ductal Hyperplasia), ADH (Atypical Ductal Hyperplasia), DCIS (Ductal Carcinoma In Situ), LIN (Lobular Intraepithelial Neoplasia).

## Data Availability

The raw data supporting the conclusions of this article will be made available by the authors on request.
